# A Systematic Approach for the Interview of the Application to Psychiatry Specialty Training: The “I AM” Approach

**DOI:** 10.1192/bjo.2022.135

**Published:** 2022-06-20

**Authors:** Jiann Lin Loo

**Affiliations:** Betsi Cadwaladr University Health Board, Wrexham, United Kingdom

## Abstract

**Aims:**

Job interviews are the platform for employers to identify suitable candidates for vacant posts, i.e. those who are able to demonstrate a certain set of competencies specified in the job description. In the recent psychiatry specialty training (ST) application process, candidates are required to propose a management plan for two complex scenarios. This interview can be stressful given the high-stakes nature of the outcome, i.e. the successful enrolment into a training programme of a preferred deanery. Candidates who are unable to have an organised approach to problem-solving will likely have an unfavourable result. To overcome this difficulty, a simplified “I AM” approach is being proposed to assist applicants to organise their thoughts during their ST application interview.

**Methods:**

The “I AM” approach stands for “Issues, Assessments, and Management”, which is adapted from the “Handbook of Psychiatry: Surviving Consultation Viva Examination of Malaysian Conjoint Board”. The “Issues” are the problems identified in a scenario, “Assessments” are the investigation required to get a clearer picture of the problems, and “Management” is the action plan to solve the problems. This approach was piloted with five applicants of ST in psychiatry prior to their interview practices.

**Results:**

For a complex clinical case scenario, the “I AM” approach can be put into the matrix of 3 × 3 tables together with a biopsychosocial model to ensure the issues in different domains are explored thoroughly. Further sub-classification into necessary subheadings, including ideas, concerns, and expectations from different parties, can be included in the assessment matrix. Lastly, a management plan using a multidisciplinary team and collaborative decision-making model with the patient and family can be proposed. For a complex managerial scenario, the seven pillars of the National Health Service's clinical governance model involving different stakeholders can be incorporated into the “I AM” approach to explore problem-solving strategies from different angles. Positive reactions had been received from all five trainees (Kirkpatrick's Evaluation Model Level One).

**Conclusion:**

The “I AM” approach can be flexibly applied in different problem-solving scenarios and it works well with other models. The approach may be limited by inadequate information and a failure to prioritise. Further systematic evaluation of the effectiveness and generalisability of the “I AM” approach to other disciplines is required.

